# The Ability to Look Beyond: The Treatment of Peripheral Arterial Disease

**DOI:** 10.3390/jcm12093073

**Published:** 2023-04-23

**Authors:** Pasqualino Sirignano, Costanza Margheritini, Federica Ruggiero, Claudia Panzano, Federico Filippi, Luigi Rizzo, Maurizio Taurino

**Affiliations:** 1 Vascular and Endovascular Surgery Unit, Sant’Andrea Hospital of Rome, Department of General and Specialistic Surgery, “Sapienza” University of Rome, 00189 Rome, Italy; 2 Vascular and Endovascular Surgery Unit, Sant’Andrea Hospital of Rome, Department of Molecular and Clinical Medicine, “Sapienza” University of Rome, 00189 Rome, Italy; 3 Vascular and Endovascular Surgery Unit, Misericordia Hospital, 58100 Grosseto, Italy

**Keywords:** peripheral arterial disease, intermittent claudication, superficial femoral artery, medical management, endovascular procedures, surgical treatment

## Abstract

This paper offers a practical overview of the contemporary management of patients with peripheral arterial disease presenting intermittent claudication (IC), including clinical and instrumental diagnosis, risk factors modification, medical management, and evidence-based revascularization indications and techniques. Decision making represents a crucial element in the management of the patient with IC; for this, we think a review of this type could be very useful, especially for non-vascular specialists.

## 1. Introduction

Peripheral arterial disease (PAD) represents the most frequent manifestation of systemic atherosclerosis; epidemiological studies showed that the PAD prevalence, defined as an Ankle Brachial Index (ABI) < 0.9 among the adult population, varies between 10% and 18% in Western countries. Clinical manifestations of PAD range from asymptomatic disease to intermittent claudication (IC) and Chronic Limb Threatening Ischemia (CLTI). Approximately 30% of patients with PAD present with IC, whereas only 0.4% of patients with PAD present with CLTI [1,2,3].

The prognosis of patients with IC is mainly determined by cardiac or cerebrovascular complications, as the risk of disease progression to CLTI is low: indeed, it has been estimated that no more than 5% of patients with functional PAD will progress into CLTI, with rest pain or gangrene, while 25% have deteriorated walking distance, 50% remain clinically stable. Moreover, IC is frequently sustained by an isolated femoropopliteal district involvement, while CLTI is a consequence of a more complex arterial involvement presenting with multilevel disease extending to the infrapopliteal segment [4,5,6].

Since risk factors determining PAD overlap with those determining arterial disease in other vascular districts, patients with PAD are often at an increased risk of major adverse cardiovascular events (MACE) in addition to the major adverse limb events (MALE) risk. Therefore, the goal of the treatment for IC patients is primarily to reduce cardiovascular events rates more than reduce physical limitations and to improve daily functional abilities through symptom relief [1].

Consequently, IC due to isolated femoropopliteal lesions is more and more considered a medical disease rather than a surgical pathology. Unfortunately, the latest available PAD guidelines mainly focus on CLTI. However, the 2017 ESC-ESVS European guidelines specifically recommend risk factor control and targeted medical therapy as the cornerstone in the management of IC patients aiming to reduce cardiovascular events and mortality and to improve limb-related symptoms [7].

## 2. Diagnosis

A detailed medical record and a thorough physical exam are both essential in any patient with PAD. It is always recommended to investigate the patient’s medical history, with a special focus on lifestyle habits, diet, physical activity, walking autonomy, and familiarity history for MACE and MALE [7].

### 2.1. Physical Examination

The physical examination should include inspection, palpation, and auscultation. Upon inspection, the extremity should be evaluated for evidence of calf muscle atrophy, loss of hair growth, and thickening of the nails. Tissue loss and ulcerations must also be looked for, specifically on the toes, heels, or fingertips, paying particular attention to the interdigital area.

On initial palpation, all accessible pulses should be noted and compared with the contralateral extremity, as well as changes in temperature and sensation. The diagnostic algorithm also includes arteries auscultation, as the bruit presence might be expressive of underlying pathology.

PAD presents with a broad spectrum of signs and symptoms, beginning with intermittent claudication (defined as pain, a cramp, or sense of fatigue in a muscle group related to sustained exercise and relieved promptly by a few minutes of rest) and worsening to pain at rest and tissue loss [8].

Anatomically, depending on the location of the arterial lesion, PAD is broadly classified as aortoiliac, femoropopliteal, or tibial, and the patient may experience pain in any of the three major muscle groups of the lower extremity: the buttock, the thigh, or the calf.

Most patients present with clear and precise symptoms strongly suggestive of PAD. Nevertheless, some patients are completely asymptomatic, while others could have symptoms, especially pain, masked by the presence of other pathologies. In these cases, it is essential to recourse to further tests to eventually recognize PAD as a trigger for symptoms.

### 2.2. Non-Invasive Tests

Non-invasive testing plays a key role in PAD confirmation in patients with suggestive symptoms and differential diagnosis of other pathological conditions. The ABI is an effective and reproducible test consisting of the ratio between systolic blood pressure measured at the ankle and systolic blood pressure measured at the arm. An ABI lower than 0.90 indicates a mild PAD, between 0.90 and 0.50 indicates a mild to moderate disease, and lower than 0.50 is suggestive of severe PAD associated with a high risk of CLTI and limb loss. Notably, pathological falsely elevated values above 1.3 are often found in patients with diabetes or renal insufficiency because of the lack of arterial wall compliance due to extensive calcifications.

In the case of suspected PAD with normal ABI, pathological pressure differences can be triggered by physical exercise, with the detection in such cases of a reduction in ABI of at least 20% over the value measured at rest [7].

### 2.3. Imaging

*Duplex Ultrasound (DUS)* represents, nowadays, an essential tool for PAD assessment. It is non-invasive, cost-effective, and suitable for sequential examination. Indeed, DUS allows proper disease definition and evolution in response to physical, pharmacological, or invasive treatment. In most cases, DUS provides enough data to indicate a proper treatment, without the need for further invasive investigations. Nevertheless, DUS presents some drawbacks, including reduced accuracy in patients with heavily calcified arteries or in obese patients, and unfortunately, DUS accuracy is strictly related to operators’ experience because their knowledge and skills could affect exam reliability. Lastly, although their use is still restricted, recent techniques such as flow imaging and live 3D echography, as well as the use of ultrasound contrast agents, further enhance DUS performance [9,10].

*Computed Tomography Angiography (CTA)* offers several advantages in the diagnosis of PAD, including fast acquisition, widespread access, high degree of resolution, and 3D image reconstruction. Compared to DUS, CTA provides a “roadmap” of vascularization, which is crucial when an interventional strategy is planned. However, the acquisition of functional and hemodynamic data is not permitted by CTA, and the exposure to radiations as well as the need for iodinated contrast agents, are not negligible limitations [11].

*Magnetic Resonance Angiography (MRA)* via contrast and non-contrast image acquisition techniques represents a valuable alternative in patients with mild to moderate chronic kidney disease. MRA achieves better soft tissue resolution than CTA while requiring no iodine contrast; on the other hand, motion artifacts are more common. Additionally, this technique is not suitable for people who have claustrophobia, serious kidney disease, pacemakers, or implantable cardioverter defibrillators. MRA tends to overestimate the degree of stenosis in individuals with PAD despite its high sensitivity and specificity, and since arterial calcifications are invisible in MRA, estimating the degree of stenosis in heavily calcified lesions can be challenging [12].

## 3. Treatment

### 3.1. Modification of Risk Factors

*Smoking Cessation:* Smoking doubles the risk of PAD; it has been estimated that almost 50% of PAD diagnoses are related to smoking habits, and smoking cessation determines a significant improvement in functional outcomes, better post-operative outcomes, and overall reduction in MALE, MACE, and death occurrences [13,14,15,16,17]. Therefore, any effort on smoking prevention and cessation consisting of medical advice, group therapy sessions, and nicotine replacement should be offered to all smokers [18]. The use of bupropion has received support from several randomized studies, and its combination with nicotine replacement treatment has been shown to be more effective than either medication alone [19,20].

*Supervised Exercise:* Published studies showed that supervised treadmill exercise and home-based exercise that incorporate behavioral change techniques significantly improve pain-free and maximal walking distance in IC patients [21,22,23]. Regular walking activity (3–5 times per week) improves walking performance in 4–6 weeks after the beginning of supervised/home-based exercise. Although data suggest that walking until ischemic leg pain begins is beneficial, effective exercise programs for PAD patients recommend walking until near maximal leg pain [24,25]. Home-based training is an effective alternative to exercise-supervised programs in terms of walking distance improvement and has proven to be more accessible and well tolerated by patients with PAD, not requiring traveling to an exercise center [26].

*Dyslipidemia:* Elevated levels of total cholesterol, low-density lipoprotein cholesterol (LDL-C), triglycerides, and lipoprotein(a) are all independent risk factors for PAD, and dyslipidemia is one of the main risk factors affecting PAD prognosis. Recent studies have shown that lowering LDL-C is linked to MACE and MALE occurrence reduction, especially for high-risk patients [27,28]. ESVS guidelines recommend a target serum LDL-C < 70 mg/dL or decreased by >50% if the initial LDL-C level is between 70 and 135 mg/dL [7]. Available data also provide clear support for the use of statins to reduce LDL-C cholesterol levels in PAD patients: daily statins administration showed a 22% relative risk decrease in the first major vascular event in PAD patients and a 78% reduction in MACE compared to placebo [29]. Moreover, several studies have revealed the beneficial effects of statin therapy in improving walking distance, or alternatively in slowing down its decline, in IC patients with intermittent claudication [30,31]. Nevertheless, since PAD patients typically already experience leg pain, the issue of myalgias and statin-induced myopathy is not negligible. Due to the various pharmacokinetic and pharmacodynamic characteristics of different commercially available statins, their efficacy and side effects may vary significantly. Since cardiovascular benefits outbalance any risks in PAD patients, in case of muscular pain from a statin, a lower dose of the same statin or a different one should be prescribed [32].

*Arterial Hypertension:* Appropriate blood pressure control reduces the risk of cardiovascular events and associated mortality [33,34]. The European Society of Hypertension guidelines state that blood pressure target values below 140/90 mmHg, apart from diabetic patients who should have a diastolic blood pressure lower than 85 mmHg [35]. Diuretics, beta blockers, calcium antagonists, angiotensin-converting enzyme inhibitors (ACEIs), and angiotensin receptor blockers (ARBs) are all suitable for antihypertensive treatment (as monotherapy or in different combinations). Notably, ACEIs and ARBs are recommended as first-line therapy because both demonstrated a significant reduction in MACE events in patients with PAD, without any impact on limb outcomes [35].

*Diabetes:* In patients with diabetes, PAD is particularly frequent and has a worse prognosis than in patients without diabetes, with a two-fold risk of mortality and a four- to five-fold risk of lower-limb amputation [36]. Moreover, in patients with type 2 diabetes, PAD is two to three times more prevalent than in the general population, and the prevalence also increases with the duration of diabetes, as shown by the UK Prospective Diabetes Study and by the Western Denmark Heart Registry [37,38].

Although epidemiological studies and randomized controlled trials have shown the benefit of intensive glycemic control in reducing diabetic-related long-term microvascular complications, the efficiency of intensive glycemic control in the prevention of cardiovascular disease, including PAD, and death has not been established [39,40]. Nevertheless, available guidelines suggest a glycated hemoglobin (HbA1C) goal of <7% (53 mmol/mol) to reduce the risk of MACEs in diabetics [41,42].

Notably, guidelines do not specifically mention management for PAD, but for atherosclerotic disease in general, suggesting that the medical management of PAD in patients with diabetes is not different from that recommended for all other atherosclerotic patients. Nevertheless, both documents recommend among patients with type 2 diabetes who have established atherosclerotic cardiovascular disease, a Glucagon-like peptide-1 (GLP-1) receptor agonist or sodium–glucose co-transporter 2 (SGLT-2) inhibitor as part of the comprehensive cardiovascular risk reduction [41,42].

### 3.2. Medical Management

The goal of medical management for IC patients is to reduce physical limitations, relieve symptoms, and improve quality of life. Over time, in addition to the development of therapies aimed at controlling risk factors, data have emerged promoting the routine use of anticoagulants and antiplatelet drugs in IC patients.

ESVS guidelines support long-term single antiplatelet therapy for symptomatic patients to reduce MACE and death occurrences, while no benefit is reported for asymptomatic PAD patients [7].

Regarding antiplatelet drugs to be administered, guidelines suggest the use of clopidogrel rather than Acetyl Salicylic Acid (ASA) [7]. The CAPIRE study (Clopidogrel versus Aspirin in Patients at Risk of Ischemic Events) showed a significant reduction in the combined risk of ischemic stroke, myocardial infarction, or vascular mortality by long-term therapy with clopidogrel versus ASA. Interestingly, ticagrelor was not superior to clopidogrel in PAD patients [43,44]. Lastly, dual antiplatelet therapy is not indicated in patients with PAD, apart from those patients with a positive medical history of myocardial infarction, given the increase in bleeding [45,46].

Because some PAD patients have a high risk of atherothrombotic complications despite antiplatelet therapy, anticoagulant-based therapeutic strategies have been proposed to reduce cardiovascular morbidity or mortality in those patients. Unfortunately, the WAVE (Warfarin Antiplatelet Vascular Evaluation) trial failed to show a reduction in MACE rate for patients treated with ASA plus Warfarin, compared to those treated with single antiplatelet therapy alone, while the double antiplatelet therapy was related to a significant excess of moderate or life-threatening bleeding events [47]. Therefore, current guidelines do not support oral anticoagulants in PAD patients, except in the case of specific indications such as atrial fibrillation, mechanical valve implantation, or other medical condition requiring an anticoagulant by itself [7].

Notably, novel oral anticoagulants (NOACs) have demonstrated very promising results. According to the COMPASS (Cardiovascular Outcomes for People Using Anticoagulation Strategies) study, patients with PAD who received Rivaroxaban 2.5 mg twice daily plus ASA (100 mg/day) of ASA experienced a 28% reduction in MACE, a 46% reduction in MALE, and a 31% reduction in the composite endpoint occurrence rates compared to ASA, with no excess in fatal or critical bleedings [48]. Similarly, the Vascular Outcomes Study of ASA Along with Rivaroxaban in Endovascular or Surgical Limb Revascularization for PAD (VOYAGER PAD) showed a significant reduction in MACE and MALE occurrences after revascularization in patients treated with Rivaroxaban plus ASA compared to patients treated with placebo +ASA [49].

Obviously, the rationale for the use of statins, antihypertensives, antiplatelets, and anticoagulants is the reduction in overall cardiovascular mortality; consequently, different vasoactive drugs have been proposed to improve patients’ quality of life, to reduce symptoms, and to improve walking distance in IC patients. Among them, Cilostazol is a phosphodiesterase-3 inhibitor that increases intracellular cyclic adenosine monophosphate (cAMP) levels, leading to the inhibition of platelet aggregation and vasodilation. Its efficacy was demonstrated over 20 years ago in a meta-analysis of eight studies which reported a 50% increase in maximal walking capacity of 50%, and a 67% increase in pain-free walking distance, when compared to the placebo [50]. Because these results were also confirmed by a recently published meta-analysis [51], guidelines recommend Cilostazol (100 mg twice daily, for at least six months) as an effective treatment to improve symptoms and increase walking distance in IC patients [52]. Cilostazol presents several generally well-tolerated side effects, such as headache, palpitations, dizziness, and gastrointestinal complaints, but its potential contraindications in patients with heart failure are controversial [53,54,55,56].

Other vasoactive drugs, such as pentoxifylline and naphthidrofuryl oxalate, while demonstrating improvement in symptoms, have not shown significant long-term benefits compared to Cilostazol. Nowadays, they should only be proposed to patients with specific contraindications to Cilostazol assumption [57].

### 3.3. Surgical and Endovascular Management

Management of IC patients has been dramatically challenged over the past two decades. Nowadays, revascularization should be limited to cases where, even after supervised exercise and optimal medical management, disabling symptoms significantly affect daily life activities [7].

According to Steiner and Schmidt, endovascular treatment techniques have dramatically changed over the past 20 years. New products, technologies, and procedural techniques allow us to successfully treat even complex lesions by endovascular techniques, and nowadays, most patients can be minimally invasively treated in line with an “endovascular first” strategy (Table 1). Nevertheless, bypass surgery remains an important option for patients with advanced disease. The techniques used vary depending on the clinical presentation, location, and complexity of the lesion [58].

*Plain Old Balloon Angioplasty (POBA):* was the earliest method of percutaneous transluminal angioplasty (PTA). It has high intra-operative success rates, ranging from 98% to 100% [59], but it is linked to several acute complications such as residual stenosis, vessel recoil, and flow-limiting dissections, for which bailout stenting is often required. According to the Trans-Atlantic Inter-Society Consensus document (TASC II) [59], PTA should be performed in case of stenosis/occlusion < 100 mm, especially in no stent-preferred zones, given the need for stent placement only in case of intra-procedural acute failure. Due to the necessity for long-term patency, which PBA frequently does not provide, there has been a significant development in angioplasty-based interventions.

*Stents:* Due to the constant presence of strong biodynamic forces, such as longitudinal and radial compression, flexion, extension, and torsion, which cause tension on the vessel walls, the femoropopliteal vascular axis is one of the most challenging districts to perform surgical and endovascular procedures. Despite the necessity to use stents to overcome immediate POBA failures and the reported better acute results in lesions longer than 100 mm [60], most products for this specific area have provided unsatisfactory results, likely because of their inadequate anatomical and physiological features. Furthermore, kinking and fractures typically occur in repeated-stressed areas, and restenosis and neointimal hyperplasia in zones where the stent’s radial force is maximal [61,62]. A variety of laser-cut nitinol stents have been developed with the evolution of stent design and delivery systems to optimize radial strength, flexibility, and implantation accuracy. This trend has led to the development of biomimetic stents, such as Supera stents (Abbott Vascular, Santa Clara, CA, USA). This self-expandable stent, specifically designed for the femoropopliteal district, is a braided inter-woven nitinol device that confers higher radial strength while enabling the stent to flex and bend, giving it the capacity to mimic the normal arterial movements of the leg during hip and knee flexion. Considering, however, the importance of adequate preparation of the target vessel and the achievement of an optimal deployment technique, many studies have demonstrated that the Supera stent can be considered a valid option for primary stenting [63,64,65,66].

*Vessel Preparation:* The latest trend in the endovascular field is attempting to perform femoropopliteal revascularization “leaving nothing behind”, and avoiding stent deployment. Consequently, several devices have been developed to properly manage the plaque and to prepare the vessel for subsequent treatments, improving short- and long-term outcomes through plaque removal or modification, and enhancing drug deposition into the vessel wall. The more commonly used devices are briefly described as follows. *Atherectomy* devices debulk heavily calcified lesions, improving drug delivery and promoting full-vessel expansion. Based on their mechanism of action, atherectomy devices can be divided into four classes: (1) orbital atherectomy that consists of an eccentrically mounted diamond-coated crown; (2) rotational atherectomy that is equipped with concentrical front-cutting blades; (3) directional atherectomy that is indicated for eccentric plaques thanks to its side-cutting blades; and (4) laser atherectomy that employs radiation to fragment the atheroma [67]. Regardless of mechanical action, all these devices carried out a certain risk of plaque debris migration and consequent distal embolization. The amount of embolized material may be limited by new-generation devices that offer aspiration in addition to atherectomy capabilities, but to minimize embolic events, distal filters should be considered, as well as vasodilators to prevent vasospasm [68]. *Special Balloons:* Different from POBA, the primary goal of these devices is to improve vessel compliance, lumen gain, and drug delivery [69]. Scoring balloons are semi-compliant balloons covered by helical scoring elements or longitudinal wires useful for a precise rupture of the plaque; cutting balloons have longitudinally oriented sharp metal blades on their surface to cut the atherosclerotic plaque. Despite a dramatic effect on plaque rupture and wall remodeling, both devices carried out a significant risk of acute dissection or arterial wall perforation. To maximize vessel preparation and reduce dissection rates, a new-concept device (Chocolate Balloon; Medtronic, Santa Rosa, CA, USA) was developed. This balloon is enveloped in a nitinol constraining cage, which expands with balloon expansion, protecting the vessel wall from the torsional stress during inflation. Additionally, the nitinol cage, during inflation, creates a peculiar “pillows” and “grooves” structure that act as stress relief areas, simultaneously maximizing angioplasty benefits while allowing for plaque modification and limiting dissection [6]. *Intravascular Lithotripsy:* A different method of vessel preparation, with a peculiar mechanism of action, is intravascular lithotripsy (IVL). The Shockwave intravascular lithotripsy device (Shockwave Medical, Santa Clara, CA, USA) consists of a balloon catheter containing an ultrasound generator. After inflation of the balloon, sonic waves are generated for a few seconds, resulting in microfractures of the intimate and medial calcium deposits. This technique increases vessel compliance and improves the delivery of antiproliferative drugs delivery, without the risk of distal embolization due to plaque debris migrations [70].

*Focal Self-Expanding Nitinol Stents:* Aiming to overcome stent limitation and maximize its mechanical role in residual dissections, a new stent system for spot and short (13 mm in length) stenting has been introduced for the treatment of femoropopliteal lesions. As reported, the peculiarity of having multiple short stents instead of longer ones has the advantage of reducing the mechanical strain to which the stents can be subjected by bending and stretching, especially in the distal SFA or the popliteal artery. Preliminary data are promising, with no device-related complications, no major adverse events, and a 100% patency at 6 months [71,72].

*Drug-Coated Balloons and Drug-Eluting Stents:* Furthermore, drug-coated balloons (DCBs) are available to further improve long-term outcomes of femoropopliteal revascularization, preventing restenosis and re-occlusion without the necessity of leaving metal behind. DCBs have a dual action; balloon dilatation is combined with the reduction of neointimal hyperplasia mediated by antiproliferative compounds on the surface of the balloon, among which the main one is paclitaxel. The efficacy of paclitaxel-based DCBs has been proven in several randomized clinical trials to be superior compared to conventional PTA [73,74,75], providing long-term benefits up to 5 years after revascularization [76]. Lastly, no significantly different results were achieved using paclitaxel-based drug-eluting stents (DES) compared to bare metal stents [77]. Moreover, high-dose DCB, after a successful vessel preparation, showed comparable rates with DES in terms of freedom from target lesion revascularization [78]. Over time, because of controversial findings regarding the safety of paclitaxel-based DCBs [79,80], the development of devices led to the introduction of Sirolimus as a new coating antiproliferative drug. Sirolimus inhibits lymphocyte activations and smooth muscle and endothelial proliferation and, compared to paclitaxel, is related to higher tissue tolerance in terms of vasculotoxicity. The safety and feasibility of Sirolimus-coated balloons are demonstrated by preliminary findings [81,82]. However, to draw more certain conclusions on the patency and procedural effectiveness of this type of balloon in the endovascular treatment of peripheral artery disease, more investigation and larger sample sizes will be essential.

*Advanced Endovascular Procedures:* Several other procedures are technically available in femoropopliteal revascularization, such as endovascular bypass using peripheral endograft [83] (Viabahn; WL Gore and Ass, Flagstaff, AZ, USA), retrograde distal (even pedal) access [84], and deep veins arterialization [85]. However, all these procedures are complex, time-consuming, and expensive, with a non-negligible risk of acute complications. Therefore, they should be limited patients with CLTI and no conventional surgical or endovascular revascularization treatment options [85,86].

*Surgical Procedures:* In addition to all available endovascular therapy, surgical techniques, such as femoral endarterectomy and bypass, still represent a valid treatment in the particular setting of IC. In general, ESVS guidelines recommend surgical procedures in approaching femoropopliteal occlusion or stenosis longer than 25 cm [7]. Regarding the conduit selection for bypass, in the case of above-the-knee bypass, the great saphenous vein (GSV) is the gold standard, and prosthetic grafts should be considered in case of the absence or non-suitability of the vein [87,88]. A Cochrane review of 19 studies, including more than 2000 patients submitted to above-the-knee bypass, showed moderate-quality evidence that autologous GSV graft improved primary patency compared to prosthetic grafts at 60 months (*p* = 0.005), and low-quality evidence to suggest this benefit translated to secondary patency, too (*p* = 0.003). No clear difference was found between Dacron and Polytetrafluoroethylene (PTFE) grafts for primary patency at 60 months, while low-quality data showed better secondary patency for Dacron grafts over PTFE (*p* = 0.005) [89]. Lastly, although GSV is considered the best conduit for below-the-knee bypass [90], the review, analyzing results on 576 performed interventions, showed no graft type to be superior to any other in terms of primary patency [89].

*Financial Implications:* It is not simple to standardize procedural-related costs for surgical or endovascular revascularization for IC patients with femoropopliteal occlusion. This difficulty is strictly related to differences in terms of cost evaluation (direct, indirect, immediate, long-term, and quality-of-life-related costs), and to the plethora of available revascularization techniques. Nevertheless, Vossen and co-workers recently published a very elegant comparative cost-effectiveness analysis of endovascular and surgical revascularizations for medium-length femoropopliteal lesions [86]. Over a period of 3 years, they collected all the hospital costs for 226 consecutive patients (135 treated by endovascular procedures and 108 by surgical bypass). The main measure of outcome was the primary patency rate at 3-year follow-up, and the adjusted incremental cost-effectiveness ratios (ICERs), calculated as the ratio between the difference in total costs and the difference in 3-year primary patency rate. In their experience, mean total costs per patient were EUR 29,058 in the endovascular group vs. EUR 42,437 in the surgical group; the difference in 3-year primary patency between endovascular and surgical procedures was small and nonsignificant (68.9% and 70.3%, respectively). An ICER of 563,716 was found, indicating that surgery cost EUR 563 more per each extra patient reaching 3-year primary patency in comparison with endovascular treatment. Accordingly, they concluded that surgery cost higher than endovascular treatment over a 3-year follow-up period [86].

## 4. Conclusions

IC is one of the most frequent and clinically relevant PAD manifestations. Most of the patients, fortunately, present few symptoms, without severe claudication limiting their quality of everyday life. Consequently, most IC patients can (and should) be managed with risk factors correction and control, physical exercise, and the best medical therapy.

In the few patients who require intervention (surgical or endovascular), the choice of the right technique is never a simple operation and, therefore, must be faced by expert vascular surgeons skilled in both surgical and endovascular techniques.

## Figures and Tables

**Table 1:** Advantages and disadvantages of all endovascular technologies available for femoropopliteal lesion treatment in IC patients.

	Advantages	Disadvantages
**Plain Old Balloon Angioplasty**	High immediate technical success, easy to use, efficacious when treating lesions < 100 mm	High rates of flow-limiting dissections, vascular recoil, and residual stenosis High restenosis rate
**Atherectomy**	As vessel preparation, prior to plain balloon or drug-covered balloon angioplasty, reduces inflation pressures and related risk of dissections	Risk of distal embolization High procedural cost Lack of randomized data and limited prospective data
**Special Balloons**	As vessel preparation, prior to plain balloon or drug-covered balloon angioplasty, reduces inflation pressures and related risk of dissections	Lack of randomized data and limited prospective data
**Intravascular Lithotripsy**	As vessel preparation, prior to plain balloon or drug-covered balloon angioplasty, reduces inflation pressures and related risk of dissections	Lack of randomized data and limited prospective data
**Stents, Drug-Eluting Stents**	Improved patency when treating lesions > 100 mm	High rates of restenosis (with long lesions >200 mm), material fatigue, and stent fractures For Drug-Eluting Stents, concerns about paclitaxel safety
**Focal Self-Expanding Nitinol Stents**	Mechanical effect is limited to dissection sites only. Lower rates of restenosis material fatigue and stent fractures, with respect to conventional stents	Limited prospective data
**Drug-Coated Balloons**	Improved long-term patency compared with results achieved by plain balloons	If used alone, same rates of flow-limiting dissections, vascular recoil, and residual stenosis as plain balloons Concerns about paclitaxel safety

## Data Availability

Not applicable.
